# Systematic and comprehensive insights into HIF-1 stabilization under normoxic conditions: implications for cellular adaptation and therapeutic strategies in cancer

**DOI:** 10.1186/s11658-024-00682-7

**Published:** 2025-01-06

**Authors:** Jiayi Zhang, Mingxuan Yao, Shiting Xia, Fancai Zeng, Qiuyu Liu

**Affiliations:** 1 School of Pharmacy, Southwest Medical University, Luzhou, 646000 China; 2 Laboratory of Biochemistry and Molecular Biology, School of Basic Medical Science, Southwest Medical University, Luzhou, 646000 China; 3 School of Clinical Medicine, Southwest Medical University, Luzhou, 646000 China

**Keywords:** HIF-1, Normoxia, Pseudohypoxia, Mechanisms, Cancer, Cellular response, Target therapy

## Abstract

Hypoxia-inducible factors (HIFs) are essential transcription factors that orchestrate cellular responses to oxygen deprivation. HIF-1α, as an unstable subunit of HIF-1, is usually hydroxylated by prolyl hydroxylase domain enzymes under normoxic conditions, leading to ubiquitination and proteasomal degradation, thereby keeping low levels. Instead of hypoxia, sometimes even in normoxia, HIF-1α translocates into the nucleus, dimerizes with HIF-1β to generate HIF-1, and then activates genes involved in adaptive responses such as angiogenesis, metabolic reprogramming, and cellular survival, which presents new challenges and insights into its role in cellular processes. Thus, the review delves into the mechanisms by which HIF-1 maintains its stability under normoxia including but not limited to giving insights into transcriptional, translational, as well as posttranslational regulation to underscore the pivotal role of HIF-1 in cellular adaptation and malignancy. Moreover, HIF-1 is extensively involved in cancer and cardiovascular diseases and potentially serves as a bridge between them. An overview of HIF-1-related drugs that are approved or in clinical trials is summarized, highlighting their potential capacity for targeting HIF-1 in cancer and cardiovascular toxicity related to cancer treatment. The review provides a comprehensive insight into HIF-1’s regulatory mechanism and paves the way for future research and therapeutic development.

## Introduction

Hypoxia-inducible factors (HIFs) are critical transcription factors that mediate cellular responses to oxygen deprivation. Under normal oxygen conditions (normoxia), HIFs are usually hydroxylated by prolyl hydroxylase (PHD) domain enzymes, which modify HIFs for ubiquitination and subsequent degradation by the proteasome, ensuring that their levels remain low [1]. However, during hypoxic stress, reduced oxygen availability impairs PHD activity, leading to the stabilization and accumulation of HIFs.

The HIFs are transcription factors composed of an oxygen-sensitive subunit and a constitutively expressed subunit, which function as heterodimers. In humans, there are three distinct members of the HIF family: HIF-1, HIF-2, and HIF-3. Although the HIF-1 subunit is the most notable of the hypoxia-inducible factors, it has been demonstrated that HIF-2 also plays a role in regulating hypoxia-responsive genes in chronic hypoxia instead of acute hypoxia, which is different from HIF-1 [2]. Even though HIF-1 and HIF-2 are structural similarity, sharing a 48% identical amino acid sequence, numerous studies employing various methodologies indicate that these two factors have distinct functions and gene targets [2, 3]. In contrast, HIF-3 has different amino acid sequences compared with HIF-1 and HIF-2. Notably, HIF-3 is regarded as the negative regulator of the target genes of HIF-1 and HIF-2 [4]. The HIF-1α subunit is the most prominent, playing a crucial role in cancer development. The stabilized HIF-1α then translocates into the nucleus, where it dimerizes with HIF-1β (ARNT, an oxygen-insensitive and conservative subunit) to form active HIF-1 complexes [5]. These complexes bind to hypoxia-responsive elements (HREs) in the promoters of target genes, initiating the transcription of genes involved in adaptive mechanisms. These target genes drive essential processes such as angiogenesis (e.g., through vascular endothelial growth factor (VEGF) induction), metabolic reprogramming (including shifts to glycolytic pathways), and cellular survival (by enhancing stress resistance and modulating apoptosis) [5, 6]. This intricate regulatory mechanism enables cells to adapt to hypoxic conditions and maintain homeostasis, playing a vital role in both physiological responses to oxygen deprivation and pathological conditions, particularly in cancer.

**Fig. 1:**
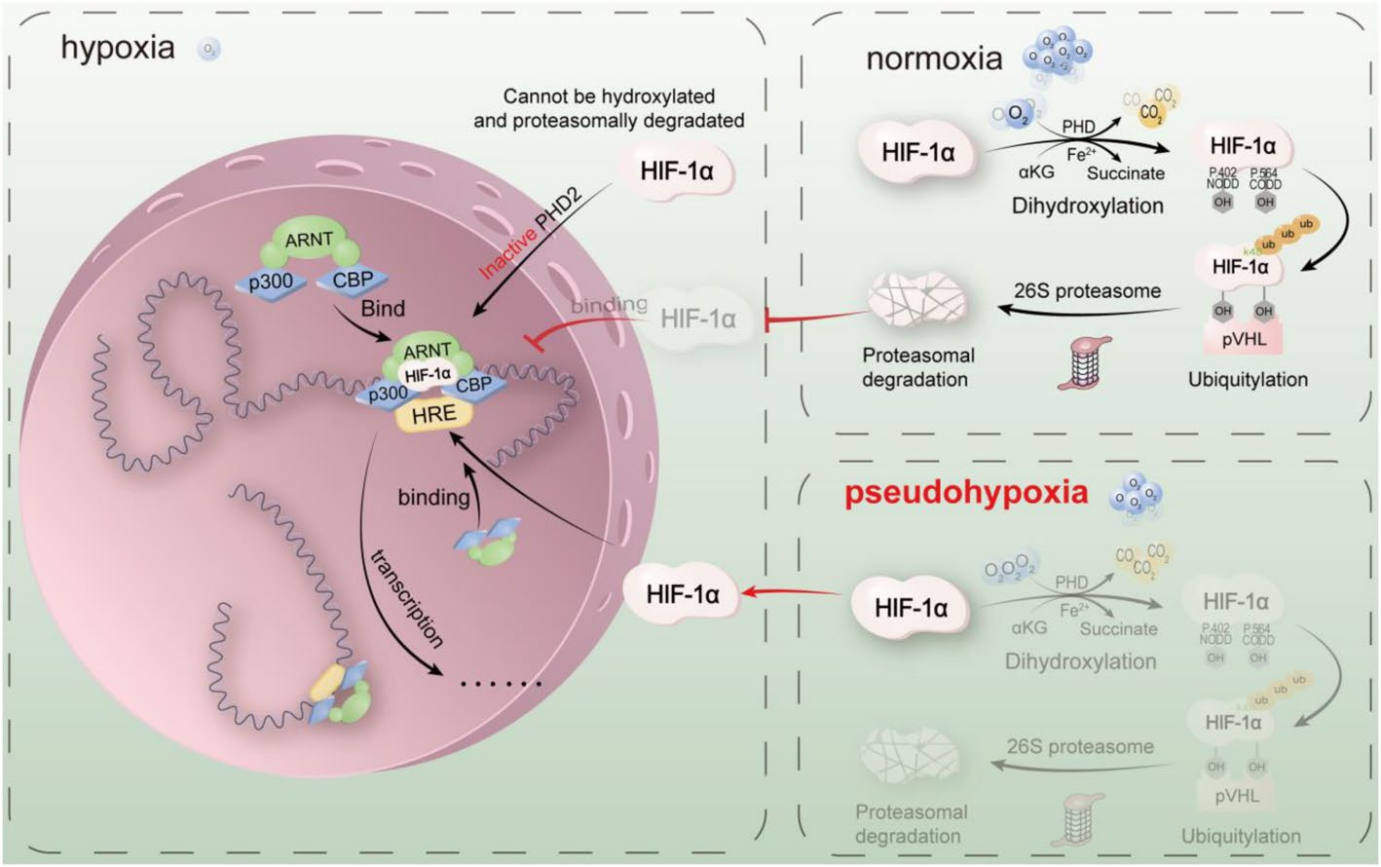
Stability of HIF-1 in normoxia, hypoxia, and pseudohypoxia under varying oxygen levels. (i) Normoxia. HIF-1α is hydroxylated by PHD2, which leads to its ubiquitination and subsequent degradation by the proteasome, resulting in being unable to initiate transcription and regulate downstream genes. (ii) Hypoxia. HIF-1α cannot be hydroxylated by PHD2 nor be degraded by the proteasome. Consequently, HIF-1 can effectively perform its functions by binding to HIF1β in the transcription of downstream genes. (iii) Pseudohypoxia. HIF-1 remains stable and continues to regulate the transcription of downstream genes, even in the presence of oxygen

However, HIF-1α can also remain stable in normoxia in malignant cancer cells or after some treatment, namely pseudohypoxia, which is defined as a phenomenon in which many cancer cells have developed the ability to activate and utilize hypoxia-dependent pathways even in the presence of oxygen [7]. The Warburg effect was regarded as an example of pseudohypoxia in research [7]. For example, HIF-1α was reported to remain relatively high even in a richly vascularized (oxygen rich) microenvironment in clear cell renal cell carcinoma (ccRCC, sporadic ccRCC cases are often caused by aberrations in the *VHL* (encode von Hippel–Lindau tumor suppressor protein) gene). The stabilized HIF-1α activates the expression of VEGF, leading to rich vascularization, which seems like a “vicious cycle,” then resulting in worse patient outcome and malignancy signs [8, 9]. The statuses of HIF-1 under different circumstances are shown in Fig. 1.

In this review, we describe the cascading and progressive mechanisms by which HIF-1α remains stable under normoxia, including but not limit to transcription, translation, and protein stabilization levels. In addition, we highlight the mechanisms associated with protein level stabilization and targeted therapeutic applications in cancer and cardio-oncology syndrome. Meanwhile, the relationship between HIF-1α stabilization under normoxia and metabolic reprogramming of tumor cells is discussed, and future development in cancer is prospected.

## Hypoxia and HIFs

Almost all animals require oxygen for survival. During growth and development, oxygen acts as an essential electron acceptor in various biochemical reactions involved in energy metabolism, playing a crucial regulatory role at the organ, tissue, cell, and molecular levels [10]. When oxygen is limited, a condition known as hypoxia, most animals respond through a specific signaling pathway involving HIFs [11].

### HIFs’ origins

**Fig. 2:**
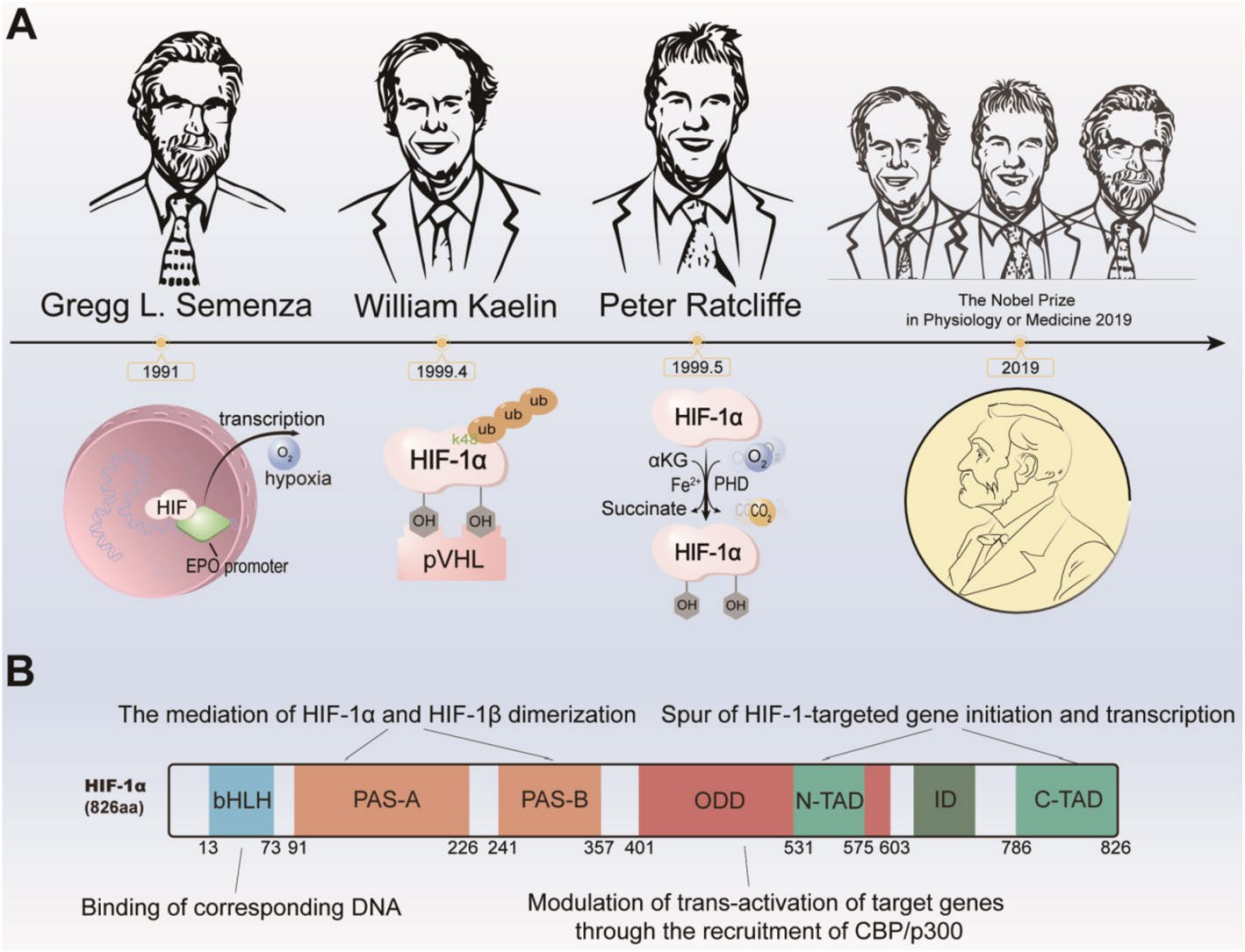
Origin and structural characteristics of HIF-1. **A** The discovery of hypoxia-inducible factors (HIFs) resulted in the award of the 2019 Nobel Prize in Physiology or Medicine to Gregg L. Semenza, William Kaelin, and Peter Ratcliffe. **B** HIF-1α possesses five functional domains serving distinct roles, including the bHLH domain, the N-TAD and C-TAD domains, the ID domain, the PAS-A and PAS-B domains, as well as the ODD domain

HIFs were originally discovered by Gregg L. Semenza in the early 1990s as newly identified proteins that bind to the erythropoietin (EPO) promoter and mediate the transcriptional induction during hypoxia [12–14]. Subsequently, William Kaelin confirmed that HIFs are targeted for destruction by von Hippel–Lindau tumor suppressor protein (pVHL) in the presence of oxygen [15], while Peter Ratcliffe identified the key regulatory components involving the oxygen-sensing regulation mechanism of HIFs, namely PHDs [16]. Together with Semenza, their discovery was awarded the Nobel Prize for Physiology or Medicine in 2019 [17]. HIFs were initially discovered as the EPO promoter, and it used to be thought of as HIF-1. However, HIF-2 was finally determined as the principal transcription factor of the EPO gene with deep research later [3, 18]. The discovery history of HIFs is shown in Fig. 2A.

Metabolic characters have always been mentioned in the bible (*Hallmarks of Cancer*) of oncology scientists since 2011, which emphasizes the importance of deregulating cellular metabolism in the progression of cancer [19, 20]. Among the HIF family, HIF-1 (rather than HIF-2 and HIF-3) plays an important role in metabolic regulation, as plentiful downstream genes of HIF-1 are highly related to the glucose metabolism process, such as *GLUT1*, whose transcription and translation products participate in the intake of glucoses [21–23]. In addition to glucose transport and angiogenesis, HIF-1 is involved in other process such as erythropoiesis, glycolysis regulation, and acidosis regulation [24]. Therefore, the importance of HIF-1 depends on its capacity to mediate the adaptive responses to oxygen concentrations [25].

### HIFs’ structural basis for stabilization

HIF-1 is composed of HIF-1α (an oxygen-sensitive subunit) and HIF-1β (ARNT) [26, 27]. HIF-1 activates gene transcription by binding to the core DNA sequence 5′-RCGTG-3′ (R = A or G) embedded in the HREs [28]. HIF-1 protein consists of a total of five utilitarian and critical types of conserved domains (Fig. 2B), including: (1) a DNA-binding (basic helix–loop–helix, bHLH) domain, (2) N-terminal and C-terminal oxygen-dependent degradation domain (NODD and CODD) [29, 30], (3) protein–protein interaction and dimerization (Per–Arnt–Sim, PAS-A and PAS-B) domain [27, 31], (4) transcriptional activation domain (N-terminal and C-terminal transactivation domain, N-TAD and C-TAD) [31, 32], and (5) a repressive domain (inhibitory domain, ID). Each domain has its respective special function. The bHLH domain plays its role in DNA binding. The N-TAD and C-TAD domains spur the HIF-targeted gene initiation and transcription process, while the ID (located between N-TAD and C-TAD) decreases the transcriptional activity of N-TAD and C-TAD. In addition, the C-TAD can modulate trans-activation of target gene through the recruitment of CBP/p300 (transcriptional coactivator) [33, 34]. The PAS-A and PAS-B domains are responsible for the dimerization of HIF-1α and HIF-1β [27]. The ODD is the pivotal oxygen-sensing domain, which is specifically possessed by HIF-1α but lacking in HIF-1β (which may explain why HIF-1β is an oxygen-insensitive subunit on a structural basis) [30, 35].

HIF-1α is the main regulator under different oxygen conditions, since HIF-1α has a short half-life and is regulated by oxygen, whereas HIF-1β remains relatively constant in different types of oxygen environments [1, 36]. HIF-1 also plays an adaptive regulatory role in cardiovascular diseases, due to myocardial infarction and other cardiovascular blockage, resulting in local myocardial hypoxia [5]. Moreover, one study found that HIF-1α conditional knockout mice rapidly developed cardiac hypertrophy and heart failure under pressure overload conditions. This finding indicates that HIF-1α plays a crucial protective role for the myocardium during the development of hypertrophy [37]. Simultaneously, high expression of HIF-1α is associated with higher mortality in multiple cancers, such as breast cancer and hepatocellular carcinoma [38]. For instance, HIF-1α is a key regulator and plays a key role through signal transduction in the process of cancer angiogenesis, metabolism, invasion, and metastasis [25, 39]. As HIF-1 is associated with cutting-edge and multiple disease medical fields, revealing its deeper mechanism is still necessary.

## Regulation of HIF-1α

**Fig. 3:**
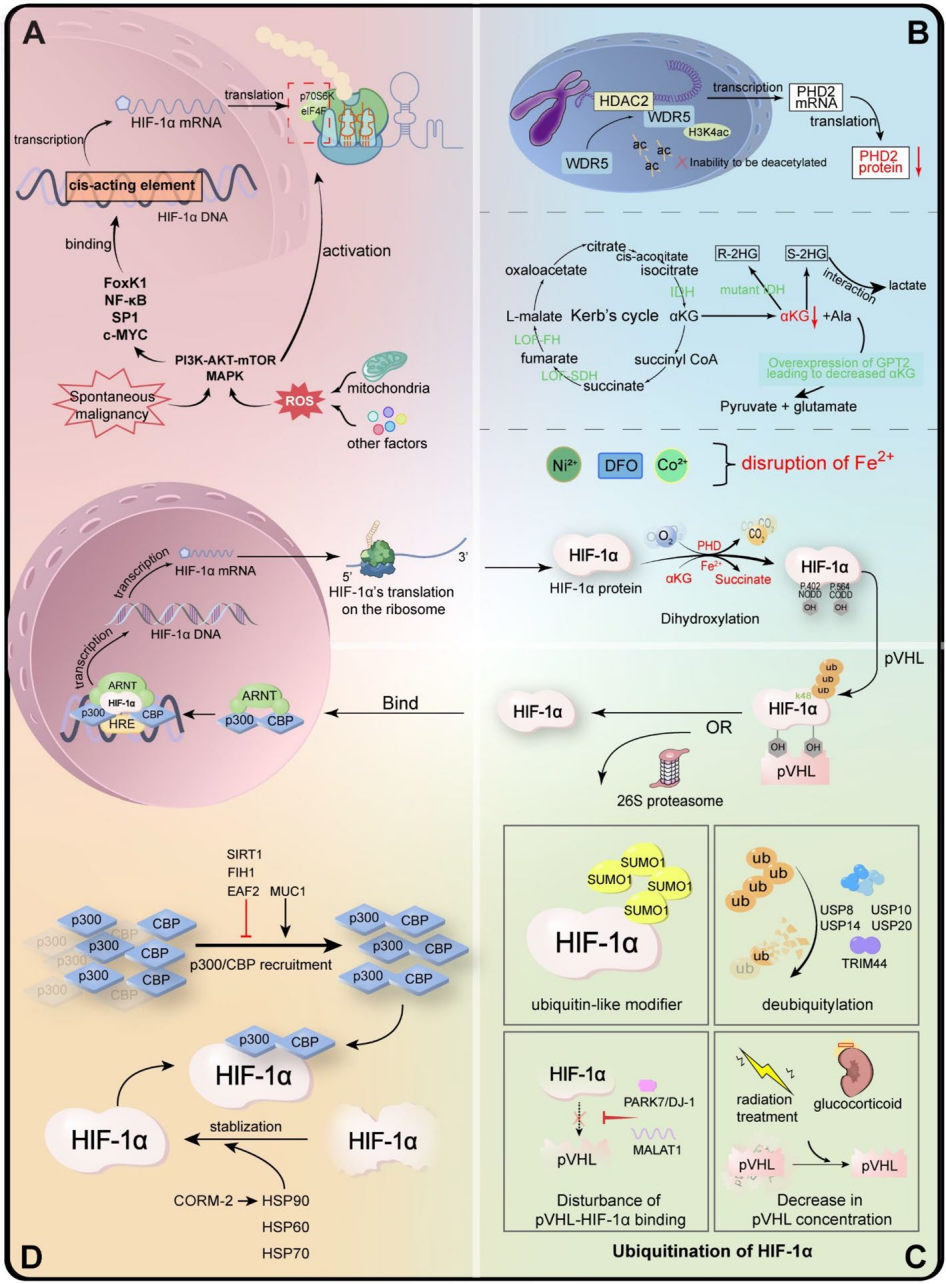
Regulatory mechanism of HIF-1 under normoxia. HIF-1α protein is synthesized through transcription and subsequent translation. Under normal oxygen levels, the newly synthesized HIF-1α protein is hydroxylated by prolyl hydroxylase 2 (PHD2) in the presence of co-activators αKG and Fe^2+^. The dihydroxylated HIF-1α is then ubiquitinated by the von Hippel–Lindau (pVHL) and degraded by 26S proteasome. However, HIF-1α protein can remain stable in pseudohypoxia. The regulatory mechanisms are outlined as follows: **A** Transcriptional and translational regulation of HIF-1α: (i) Activation of the PI3K-AKT-mTOR and MAPK signaling pathways enhance the activity of transcription factors (TFs) such as: FoxK1, NF-κB, SP1, and c-MYC, leading to increased transcription of HIF-1α; (ii) Activated PI3K-AKT-mTOR and MAPK signaling pathways also facilitate the translation of HIF-1α. **B** Stability of HIF-1α protein by modulating its PHD status: (i) Regulation of the transcription and translation of PHD2; (ii) Metabolites and associated enzymes related to αKG; (iii) Chemical compounds or environmental factors associated with Fe^2+^. **C** Ubiquitination of HIF-1α: (i) Modification by SUMO1 as ub-like modifier; (ii) Deubiquitination functions of TRIM44, USP8, USP10, USP14, and USP20; (iii) Disruption of pVHL-HIF-1α binding by proteins such as PARK7/DJ-1 and lncRNA MALAT1; (iv) A decrease in pVHL concentration induced by glucocorticoid and radiation treatment. **D** Transcriptional activation regulation of HIF-1α. Heat shock proteins HSP90, HSP60, HSP70, and MUC1 are shown to promote the stability of HIF-1α, while SIRT1, FIH1, and EAF2 are shown to reduce its stability

In this section, we discuss five dimensions of the regulation mechanism of HIF-1 from the transcriptional level, the translational level, PHD modulation, ubiquitination regulation, and transcriptional activation regulation. An overview description is shown in Fig. 3.

### Regulation of HIF-1α at the transcriptional level

In line with the central dogma of molecular biology, which underscores the fundamental importance of DNA information flow, the transcription process is essential for generating mRNA from DNA [40, 41]. *Cis*-regulatory elements, including enhancers and promoters, play a crucial role in regulating gene transcription by interacting with transcription factors (TFs). These TFs are vital for controlling gene expression at the transcriptional level [42]. The *HIF1A* gene, which encodes a protein and is located on chromosome 14q23.2, is subject to regulation by these elements. In this section, we describe the four key TFs of the *HIF1A* gene and highlight the significant role of reactive oxygen species (ROS) in the process.

#### FoxK1

FoxK proteins, encompassing FoxK1 and FoxK2, belong to the Forkhead transcription factor family. They are characterized by their conserved “fork-head” and “winged-helix” DNA-binding domains, which specifically bind to the sequence 5′-TTGTTTAC-3′ [43]. FoxK1 is ubiquitously expressed in various tissues and organs. Recent research highlights its critical role in diseases such as cancer and metabolic disorders, largely through its interaction with the mechanistic target of rapamycin complex 1 (mTORC1) [44–47]. mTORC1 is one of two distinct complexes of the serine/threonine kinase mTOR, which responds to growth factors and nutrients in mammalian cells [48–50]. Studies have shown that, when mTORC1 activity is inhibited, FoxK1 is not phosphorylated owing to suppressed GSK3 signaling, leading to reduced binding of FoxK1 with 14-3-3 proteins. Consequently, hypophosphorylated FoxK1 accumulates in the nucleus, where it enhances mTORC1-mediated metabolic reprogramming by directly upregulating HIF-1α transcription [46]. Another interesting study in cancer cell lines shows that BRCA1-IRIS accelerates tumor progression by blocking PTEN, disrupting the PI3K/AKT/GSK3β pathway. This disruption leads to HIF-1α stabilization and activation independent of PHD, even under normoxic conditions [51]. Taken together, these findings suggest that the downstream effects of the PI3K/AKT/GSK3 pathway may involve FoxK1 or other coexisting transcription factors that facilitate HIF-1α transcription [51], and further investigations are needed to explore these signaling pathways in cancer cells.

#### NF-κB

Nuclear factor kappa-light-chain-enhancer of activated B cells (NF-κb) transcription factors are pivotal in regulating immunity, stress responses, apoptosis, and differentiation. Their role extends across various signaling mechanisms, biological processes, human diseases, and potential treatments [52, 53]. Early research demonstrated that ROS levels increased by H_2_O_2_, NOX4, or thrombin can induce HIF-1α transcription by enhancing HIF-1α promoter activity. Specifically, the region −197/−188 of the HIF-1α promoter is crucial for NF-κB-mediated transcriptional activation [54]. NF-κB also has been identified as a downstream mediator in the insulin-induced upregulation of HIF-1α transcription in 3T3-L1 preadipocytes [55]. Recent studies showed that NF-κB directly bound to the HIF-1α promoter, enhancing its transcription not only under hypoxic conditions but also in normoxic environments, which has been confirmed in ischemia–reperfusion, unilateral ureteral obstruction, and sepsis-induced acute kidney injury models [56, 57]. Additionally, NF-κB activation stimulated by ROS leads to increased VEGF expression, as observed with paclitaxel treatment [58], which helps to understand the mechanism of NF-κB inhibitors, such as bortezomib and triptolide, investigated in clinical trials. NF-κB inhibitors can reduce HIF-1α transcriptional activity and are being evaluated in combination with other therapies for their potential effectiveness [59, 60].

##### SP1

Specificity Protein 1 (SP1) is one of the earliest identified transcription factors and belongs to the SP/Kruppel-like factor (SP/KLF) family [61]. SP1 features conserved zinc finger motifs in its DNA-binding domains, which recognize GC-rich (SPs) and CACC (KLFs) boxes, which allows SP1 to regulate various cellular processes, including cell proliferation, cancer metastasis, and cancer stemness [62, 63]. Research has demonstrated that insulin regulates HIF-1α via a novel transcriptional mechanism that involves ROS-sensitive activation of SP1 in 3T3-L1 preadipocytes [55]. Additionally, other studies have shown that SP1 can be activated at the transcriptional level through HIF-1α binding to its promoter under hypoxic conditions [64, 65]. These findings suggest the potential existence of crosstalk between SP1 and HIF-1α.

#### c-MYC

c-MYC is a proto-oncogenic transcription factor involved in numerous cellular processes, including DNA damage repair, cell proliferation, survival, histone modifications, and cellular metabolism. Dysregulation of c-MYC is a well-known hallmark of cancer [66, 67]. Notably, c-MYC interacts with HIF-1α to enhance glucose and glutamine metabolism, leading to metabolic reprogramming [68]. Recent research has identified that WDR5 promotes epithelial–mesenchymal transition (EMT) and metastasis in cholangiocarcinoma by enhancing HIF-1α accumulation through MYC-dependent pathways [69]. Additionally, c-MYC may promote chromatin remodeling by recruiting histone acetyltransferases (HATs) and chromatin remodeling complexes, thereby facilitating the release of RNA polymerase II pausing [70].

#### ROS-mediated transcription

ROS such as superoxide anion radicals (O_2_^−^) and H_2_O_2_ have been recognized as undesirable byproducts of the oxidative mitochondrial generation of ATP [71], which provokes oxidative stress that triggers tumorigenesis and other diseases. Moderating levels of ROS is crucial for cell survival, proliferation, differentiation, and migration, making ROS a double-edged sword in cancer contexts [72, 73] (Fig. 3A).

Under hypoxic conditions, cells sense low oxygen levels and undergo metabolic reprogramming and other adaptations. Mitochondrial ROS (mROS) are produced at Complex I and Complex III during hypoxia [74, 75]. The effects of ROS are mediated by various ROS effector proteins. For example, protein tyrosine phosphatases contain highly reactive cysteine residues in their catalytic sites, which are vulnerable to oxidation by ROS [76, 77]. PTEN and PIP1B are oxidatively inactivated by ROS, leading to enhanced PI3K/AKT signaling and its subsequent physiological effects [78, 79]. However, ROS accumulation can also occur in nonhypoxic conditions, known as pseudohypoxia. Environmental factors that are collectively referred to as the exposome can contribute to ROS buildup such as chemicals like nickel (Ni^2^⁺), cobalt (Co^2^⁺), and hard-metal particles (WC–Co) used to simulate hypoxia in laboratory experiments and shown to stabilize HIF-1α through ROS generation [80, 81]. Additionally, melatonin can counteract HIF-1α-driven aerobic glycolysis by scavenging ROS [82].

ROS trigger cellular adaptations through various signaling pathways and TFs, and kinase cascades such as the mitogen-activated protein kinases (MAPK), PI3K/Akt, and ASK1 are mentioned here [83, 84]. Correspondingly, TFs that are activated by ROS comprise Nrf2, HIF-1α, AP-1, and NF-κB [84, 85], some of which are discussed in this review as TFs binding to *HIF1A* promoter to upregulate HIF-1α at the transcriptional level.

Different signaling mechanisms can be observed in various cellular contexts. For instance, PI3K, PKC, or both collectively regulate HIF-1A transcription in lipopolysaccharide-stimulated glial cells, BCR/ABL-expressing Ba/F3 hematopoietic cells, and angiotensin II-treated vascular smooth muscle cells. In contrast, ERK and JNK are responsible for mediating lipopolysaccharide-stimulated HIF-1α mRNA, which is induced in human monocytes/macrophages and hepatoma cells, respectively. The mechanisms underlying transcription regulation are illustrated in Fig. 3A.

### Regulation of HIF-1α at the translational level

Following the transcriptional products HIF-1α mRNA, the next critical step is its translational modulation. In eukaryotic cells, the translation of mRNA is a complex, multistep process involving initiation, elongation, and termination [86]. Of these stages, the regulation of initiation is particularly important. Research from the early 2000s has demonstrated that the translation of HIF-1α is not dependent on ambient oxygen levels [87]. Thus, the translational regulation of HIF-1α independent of oxygen primarily involves the phosphorylation of ribosomal protein S6 (RPS6) and the eukaryotic initiation factor 4F (eIF4F), similar to the translational regulation of other proteins. Interestingly, both RPS6 and eIF4F are regulated by the common upstream factor mTOR [88–90]. RPS6 is an RNA-binding protein that stabilizes the ribosome by interacting with ribosomal RNA, facilitating mRNA translation. It also serves as a substrate for phosphorylation by p70S6 kinase (p70S6K) [91, 92]. Meanwhile, eIF4F, a complex comprising eIF4E, eIF4A, and eIF4G, plays a crucial role in the initiation of translation in eukaryotic cells. The eIF4E subunit directly binds to the 5′-cap structure of mRNA, thereby serving as a key factor in recruiting the 40S ribosomal subunit to the mRNA [86] (Fig. 3A).

p70S6K is the downstream effector of mTOR and can be regulated by a complicated and miscellany of signaling pathways: PI3K/Akt/mTOR and p38 MAPK, to name but a key few [93–95]. Mechanically, an early study indicated that overexpressed human epidermal growth factor receptor 2 (HER2) signaling increases the rate of HIF-1α synthesis via a translational process, subsequently mediating the expression of VEGF. This process is dependent on the activity of PI3K/Akt, highlighting the coordinating role of mTOR in the context [88]. However, the coordinating role of mTOR is unveiled in this study. Subsequent research has further elucidated the role of mTOR as a key player in various biological processes [96]. It acts as a downstream component of MAPK or PI3K/Akt and serves as an upstream regulator of p70S6K, participating in pathways related to cancer, immunity, and inflammation [96–98]. For example, the MAPK/mTOR/HIF-1α signaling axis has been implicated in TNFAIP8L2-mediated glycolytic reprogramming and inflammation [97]. Recently, our experimental data also highlighted the crucial role of p38 MAPK in breast cancer cell lines under normoxic conditions (unpublished data). These findings underscore the intricate network of signaling pathways involved in the regulation of HIF-1α and its downstream effects. The mechanisms involved in transcription regulation are illustrated in Fig. 3A.

### Stability of HIF-1α protein by modulating its PHD status

EGLN prolyl 4-hydroxylases, also known as PHDs, belong to the superfamily of 2-oxoglutarate-dependent dioxygenases (2OGDDs), which play a pivotal role at the intersection of cancer metabolism and epigenetics. These enzymes function as cellular oxygen sensors by regulating HIF-1α [99, 100]. PHDs specifically hydroxylate HIF-1α at proline residues Pro402 and Pro564 in the presence of two essential co-substrates: divalent iron (Fe^2^⁺) and α-ketoglutarate (αKG, also referred to as 2-oxoglutarate), as well as molecular oxygen (O₂) [100]. Under normoxic conditions, HIF-1α is hydroxylated by PHDs, which marks it for degradation. In contrast, HIF-1α cannot undergo hydroxylation during hypoxic conditions. The hydroxylated form of HIF-1α binds to pVHL, enabling its recognition and subsequent proteasomal degradation through pVHL’s E3 ubiquitin ligase activity [101]. Among the PHD family, PHD2 (also known as EGLN1) is the primary regulator of HIF-1α and exhibits the highest activity in response to oxygen levels [102, 103]. In this section, we discuss various factors that can modulate PHD2 activity, particularly focusing on the two co-substrates, excluding oxygen. A detailed illustration of these regulatory mechanisms is presented in Fig. 3B.

#### Metabolites and their relative enzymes related to αKG

As αKG-dependent dioxygenases, PHDs are directly influenced by factors that affect the concentration of αKG, which is a crucial intermediate in the tricarboxylic acid (TCA) cycle, also known as the Krebs cycle, and plays a key role in linking TCA cycle activity with amino acid metabolism [104]. Within the intricate TCA cycle, αKG is generated through the oxidative decarboxylation of isocitrate, a reaction catalyzed by isocitrate dehydrogenase (IDH). Additionally, αKG and glutamate are reciprocally regulated through the response of glutamic pyruvate transaminase (GPT) and glutamic dehydrogenase (GDH) [105].

#### Similar analogs of αKG

Similar analogs of αKG serve as competitive inhibitors against PHDs, which include succinate, fumarase, R-2-hydroxyglutarate (R-2-HG), S-2-hydroxyglutarate (S-2-HG), etc. Succinate and fumarate are intermediates in TCA cycle, while R-2-HG and S-2-HG as enantiomers are both byproducts with low concentration in normal cellular metabolism, while the accumulation of them greatly increased in abnormal circumstances [100].

Accumulation of succinate and fumarase is mostly related to the corresponding metabolic enzymes [106]. In normal cells, αKG is catalyzed by OG dehydrogenase and succinyl-CoA synthetase and results in the product of succinate. After that, succinate is catalyzed by succinate dehydrogenase (SDH) to produce fumarate, then via fumarase (FH) to produce malate. The normal TCA cycle is stable, and all the intermediates are in a state of flux, with no excessive accumulation. However, loss-of-function mutations in the genes encoding SDH and FH have been related to several cancers, including pheochromocytoma, paraganglioma, and renal cell carcinoma, where the upregulation of HIF-1α is associated with metabolic reprogramming, highlighting the impact of dysregulated succinate and fumarate on HIF-1α activation [107–110]. The elucidating evidence regarding the mechanisms of HIF-1α activation by dysregulated succinate was developed in further studies, in addition to the role of SDH and FH [111–114].

Accumulation of R(D)-2-HG is mostly related to the mutant IDHs, reduced from αKG [115], while accumulations of S(L)-2-HG is related to lactate dehydrogenase (LDH) in response to hypoxia, also reduced from αKG [100, 116, 117]. Mechanically, isocitrate dehydrogenases (IDHs, mainly consisting of IDH1 and IDH2 isoforms) are enzymes that convert isocitrate to αKG. Mutant IDHs lose their initial catalytic function and gain the function to produce 2HG from αKG and NADPH. A recent study showed that WT IDH2 also mediated the increasing production of 2‐HG to stabilize HIF-1α [118]. Up to now, there is seemingly no study elucidating the exact underlying mechanism of HIF-1α and S-2-HG [119]; however, S-2HG has been identified to promote HIF-1α stability in CD8 T-lymphocytes and has been shown to be mutually regulated by HIF-1α [120].

#### Lactate

Lactate is an important hallmark and a crucial metabolite signifying the metabolic reprogramming that diverts cells into glycolysis, which has been demonstrated in cellular migration, invasion, immune evasion, and radioresistance of cancer cells [121, 122]. Lactate has long been identified to upregulate the concentration of HIF-1α via the inhibition of PHDs [123, 124]; however, the underlying mechanism seems unclear. One recent study shows that lactate preconditioning promotes a HIF-1α-mediated metabolic shift partially via ROS (evidence has shown that ROS influence the catalytic activity of PHDs) from OXPHOS to glycolysis [125–128].

However, there are different explanations for the phenomena mentioned above. LDH is a tetramer that is constituted by two subunits (LDHA and LDHB), and both LDHA (converts pyruvate to lactate) and LDHB (converts lactate to pyruvate) are induced by HIF-1α [129]. This phenomenon may indicate a trans-activation of HIF-1α. In addition, more recent studies verified its role in regulating the tumor microenvironment (TME) [130]. A novel study showed an interplay between macrophages and breast cancer cells through the TME, introducing HIF-1α-stabilizing long noncoding RNA, lactate, and PHD2 [131]. This may provide an advanced insight to explain lactate’s role in HIF-1α.

#### Amino acid metabolism related to αKG

αKG is an intermediate bridges amino acid metabolism with glucose oxidation in the TCA cycle [132]. Pyruvate and glutamate are generated through the catalyzation of GPT alanine transaminase with the alanine and αKG substrates in a reversible transamination manner. Research has demonstrated that overexpression of GPT2 (a specific isoform of GPT) can lead to a reduction in intracellular levels of αKG. Decreased αKG impairs the activity of PHDs that are responsible for the degradation of HIF-1α. As a result, HIF-1α becomes more stable and accumulates in the cell, finally leading to increased stemness characteristics [133]. Cells cannot survive in isolation, and their survival and growth are heavily influenced by the surroundings, so understanding the TME is essential. Recent research has explored the interaction between HIF-1α and cysteine. Under normal oxygen levels, triple-negative breast cancer cells release glutamate, which is crucial for inducing HIF-1α in nearby cells. The glutamate secretion is sufficient to activate HIF-1α through a paracrine mechanism and inhibits the xCT glutamate-cystine antiporter, causing cysteine depletion, which leads to oxidative self-inactivation of PHD2 and ultimately HIF-1α accumulation [134]. Interestingly, αKG can be converted to glutamate through the reversible action of GDH, though there is a lack of substantial research on the effects of GDH mutations.

#### Chemical molecules or environmental factors related to Fe^2+^

Certain chemical compounds and environmental factors are commonly used to simulate hypoxia under normal oxygen levels, a condition known as pseudohypoxia, which can be induced by Co^2^⁺, Ni^2^⁺, and deferoxamine. These agents are widely recognized for their ability to mimic the effects of hypoxic conditions in various experimental settings [135, 136]. Additionally, some of these metal ions are recognized as carcinogens in environmental science [137, 138]. These environmental factors and chemical compounds are collectively referred to as “hypoxia mimetics,” being capable of inducing hypoxic response via inhibition of PHDs, resulting in the accumulation of HIF-1α [139, 140]. For metal ions, there are two mechanisms by which to compete with Fe^2^⁺: They can either bind to transport proteins such as transferrin or interfere with the regulatory proteins that manage Fe^2^⁺ levels, such as PHDs [141]. In cervical cancer, bioinformatics analysis has identified the transferrin receptor as being involved in the HIF-1α signaling pathway [142]. Cu^2^⁺ has been shown to stabilize nuclear HIF-1α under normoxic conditions, leading to increased expression of HRE-dependent reporter genes. It has been demonstrated that Cu^2^⁺ inhibits prolyl hydroxylation independently of iron concentration in in vitro studies [23]. Moreover, ROS may act as oxides to oxidize divalent iron to trivalent iron and destroy the enzymatic activity of PHDs [143].

#### The synthetic regulation of PHDs protein

PHDs can maintain their activity at the protein synthesis level even under normoxic conditions. However, PHDs typically become dysfunctional owing to the absence of the co-substrate O₂ under hypoxia. PHDs are proteins that undergo normal transcriptional and translational processes. Research showed that WDR5 bound to HDAC2, enhancing H3K4ac deacetylation on the PHD2 promoter. This reduced chromatin opening and PHD2 expression, leading to the stabilization and accumulation of HIF-1α [69, 144].

#### Others

Ascorbic acid and ascorbate-2-phosphate as unconventional co-activators of PHD are shown to decrease HIF-1 activity via its reducibility [145]. Therefore, angiotensin II [146], Co^2+^ [147], and arsenite exposure [126] mediated ascorbic acid deficiency result in HIF-1 stability. Thiamine deficiency leads to the accumulation of metabolic intermediates such as pyruvate and lactate, primarily because the enzymes dependent on thiamine become dysfunctional, resulting in the ineffective conversion of pyruvate to acetyl-CoA and subsequently increasing lactate levels [148]. Additionally, hydralazine dose-dependently inhibited PHD activity and induced nonhydroxylated HIF-1α, providing evidence for HIF stabilization specifically by inhibition of PHD enzyme activity [149, 150]. During the cell immune response, vaccinia virus infection rapidly stabilized HIF-1α, which was mediated by VACV protein C16 bound to the human oxygen-sensing enzyme PHD2 and inhibited its hydroxylation of HIF-1α under normoxia [151].

### Ubiquitination regulation of HIF-1α

The controlled regulation of protein turnover is crucial for maintaining stable cell structure and function. Approximately 30% of newly synthesized proteins in mammalian cells have a short half-life of less than 10 min and must be rapidly degraded [152]. There are three main ways to attain the protein turnover: the ubiquitin–proteasome pathway, lysosomal degradation, and autophagy [153, 154]. Ubiquitination is a highly prevalent posttranslational protein modification involving a three-step enzymatic cascade, which includes E1 (ubiquitin-activating), E2 (ubiquitin-conjugating), and E3 (ubiquitin ligase) enzymes transferring ubiquitin from its C-terminal glycine to the ε-amino group of a lysine residue on the substrate [155, 156]. Meanwhile, the reverse reaction—removal of ubiquitin modifications from substrates—is catalyzed by deubiquitinases (DUBs) [157]. The most well-known and privileged ubiquitination-related enzyme of HIF-1α is pVHL E3 ubiquitin ligase, whose loss or inactivation can lead to the formation of tumors in multiple organs, such as hemangioblastomas of the brain, renal cysts and clear cell renal cell carcinoma, etc. [158]. In addition, there are other factors concerning the modulation of HIF-1α’s ubiquitination process, such as pVHL-independent ubiquitination pathways and ubiquitin-like (Ubl) pathways. A schematic representation of this mechanism is illustrated in Fig. 3C.

#### Regulation concerning E3 ubiquitin ligase

Under normoxia, pVHL E3 ubiquitin ligase normally binds to the hydroxylated HIF-1α, directing HIF-1α for proteasomal degradation [101]. Therefore, obstruction of the HIF-1α and pVHL connecting, or reduction of pVHL efficacy may inhibit the proteasomal degradation of HIF-1α. It has been reported that protein PARK7/DJ-1 [159], lncRNA MALAT1 [160], and (possibly) HDACs [161] participate in the regulation of HIF-1α and pVHL binding. Moreover, it has been shown that glucocorticoid and radiation treatment reduce the concentration of pVHL E3 ubiquitin ligase [162, 163]. With regard to another E3 ubiquitin ligase, it is reported that loss of function p53 binds to HIF-1α, protecting HIF-1α from MDM2 E3 ubiquitin ligase, and thus results in the accumulation of HIF-1α under hypoxia [164]. Simultaneously, wide-type p53 tumor suppressor protein induces the degradation of HIF-1α, while loss of function p53 contributes to HIF-1α’s stability [164].

#### pVHL-independent ubiquitination pathways

Besides the most well-known pVHL-related pathways that regulate HIF-1α’s ubiquitination status, there exist other ways to protect the targeting HIF-1α protein from ubiquitination and thereby proteasomal degradation.

#### Deubiquitination

The process of ubiquitination is dynamic and can be reversed by the action of specialized enzymes known as DUBs [165]. Deubiquitination processes and DUBs are the counterparts against the ubiquitination cascade process, involving seven known DUB families in humans [157]. To date, over 100 DUBs have been identified in human, categorized into six families on the basis of structure and function, including ubiquitin-specific proteases (USPs), ubiquitin C-terminal hydrolases, ovarian tumor proteases, Machado–Joseph disease protein proteases, motif interacting with novel DUBs, and zinc finger USPs [166]. In accordance with the intricate and normal protein modification in ubiquitination and deubiquitination level, HIF-1α can also be specifically deubiquitylated by certain DUBs [167].

USPs, which constitute about 60% of all DUBs, are the largest and most diverse family [166]. USP20 (also known as VDU2) was early reported to mediate deubiquitination of HIF-1α and compete with the pVHL system for balance [168, 169]. Moreover, it has reported that USP14 and USP8 contribute to HIF-1α stabilization in ciliogenesis and hepatocellular carcinoma, respectively [170, 171]. However, lack of USP10 (as DUB) causes an elevation in HIF-1α under normoxia in colon cancer cells. The mechanism may depend on mTOR/S6K-mediated HIF-1α protein synthesis [172]. In addition, ubiquitin C-terminal hydrolase-L1 (UCHL1) is also reported to abrogate the pVHL ubiquitination of HIF-1α, and consequently promotes metastasis [173, 174].

TRIM44 is a member of the E3 ligase families and contains a zinc finger ubiquitin protease domain (UBP) in the N-terminal domains rather than a RING domain, which functions as a DUB [175, 176]. To date, there is convincing evidence that the function of TRIM44 is related to immune regulation, viral infection, and cancers [177]. A novel study showed that TRIM44 stabilizes HIF-1α via deubiquitination and then promotes quiescent multiple myeloma cell occupancy and survival [177]. Interestingly, further studies have shown that TRIM44 activates the AKT/mTOR signal pathway, the FOXM1–EZH2 signaling pathway, and extracellular matrix remodeling in cancer progression, DNA damage repair, and autophagy [178–183].

#### Ubl pathways: small ubiquitin-like modifiers (SUMOs)

SUMO-mediated posttranslational modification is crucial for regulating various cellular processes, including gene expression, cell cycle progression, genome integrity, metabolism, inflammation, and immunity [184]. Unlike pVHL E3 ubiquitin ligase, SUMO acts as a ubiquitin-like modifier, being shown in corresponding research and reports to have a protective function in regulating HIF-1α’s stability. It was early reported that HIF-1α is upregulated through SUMO1 modification at K391 and K477 residues, which may stabilize HIF-1α and enhance its transcriptional activity [185]. Further study indicated that Cbx4 as a SUMO E3 ligase also enhanced HIF-1α sumoylations at K391 and K477 in its two SUMO-interacting motif-dependent mechanisms [186, 187].

### Transcriptional activation regulation of HIF-1α

Under normoxia, HIF-1α barely undergoes its difficult degradation process owing to the aforementioned protective factors and remains relatively stable, thus playing its role in downstream gene transcription activity. Transcriptional activation (trans-activation) regulation of HIF-1α seems to act like the gatekeeper regulating the flux of HIF-1α downstream genes. In this section, we summarize effective processes for the transcriptional activation regulation of HIF-1α concerning the translocation and protein structural modulation, including the factors inhibiting HIF (FIHs) and co-activators p300/CBP (CREB binding protein) pathways as well as chaperone heat-shock proteins pathways. A diagram of the trans-activation mechanism is presented in Fig. 3D.

#### Recruitment of HIF-1α co-activators: p300/CBP

P300 and CBP are the main transcriptional factors related to the activation of HIF-1α-responsive genes with HAT activity [34, 188]. To elucidate, p300 and CBP are paralogous and multidomain (especially the domains in cysteine/histidine-rich1 region) proteins that serve as transcriptional co-activators by binding to the transactivation domains of a vast array of transcription factors and by binding components of the general transcriptional apparatus [34, 189]. Protein Sirtuin1 (SIRT1) is found to inactivate HIF-1α by blocking p300 recruitment and consequently repress HIF-1 target genes [190]. There are different ways to mediate the recruitment of p300/CBP, the most well-known one being mediated by FIHs.

#### FIHs pathways

FIHs are members of the 2OGDD superfamily along with PHDs, and they play different roles in the regulation of HIF-1α [191]. Regarding their 2OGDD subfamily, the PHDs belong to the prolyl hydroxylase (PH) structural family VIII of 2OGDD, while FIH belongs to the Jumonji C (JmjC) structural family VI of 2OG oxygenases [192]. More importantly, with regard to the HIF-1α structure and enzymatic functions, FIHs catalyze asparagine hydroxylation at Asn803 position, encompassed by C-TAD (a domain), while PHDs catalyze proline hydroxylation in the oxygen-dependent degradation domain (ODDD) [192, 193]. Hydroxylation of HIF-1α by FIH1 prevents its interaction with transcriptional co-activators CBP and histone acetyltransferase p300, thereby inhibiting HIF-mediated transcription [194, 195]. There seems to be no contradictory function of FIHs and PHDs, as both are responsible for degradation of HIF-1α in the presence of the three co-substrates (Fe^2+^, αKG, and O_2_). Interestingly, FIHs and PHDs are not susceptible to the same degree to the aberrant regulation of the three substrates [100] and thus may play different and individually determinant roles in response to regulating factors. FIHs have been shown to participate in inflammation, infection, angiogenesis, neuroprotection, and cancer [192, 196]. For instance, FIH1 is decreased and thus promotes HIF-1α stability after influenza A virus infection [197], which hints that development of therapies targeting inhibitors of FIHs may be necessary in time.

#### Others

SIRTs are evolutionarily conserved nicotinamide adenine dinucleotide (NAD^+^)-dependent lysine deacylases or ADP-ribosyltransferases [198]. SIRT1 is found to inactivate HIF-1α by blocking p300 recruitment and consequently represses HIF-1 target genes via binding to HIF-1α and deacetylating it at Lys674, which is acetylated under hypoxia [190, 199]. In addition, MUC1 is a type I transmembrane protein and plays a significant role in the progression of cancer, particularly pancreatic adenocarcinoma [200]. It is reported that MUC1 physically interacted with HIF-1α and p300, stabilizing HIF-1α and facilitating its recruitment to glycolytic gene promoters in a hypoxia-dependent manner [113]. ELL-associated factor 2 (EAF2) inhibition of HIF-1α activity results from the disruption of p300 recruitment, which occurs independently of FIH1 and SIRT1 [201]. Most studies are conducted in hypoxia, so exploring such mechanisms under normoxia is needed for better understanding of the Warburg effect.

#### Heat-shock protein pathways

Heat-shock proteins (HSPs, a.k.a. stress proteins) are ubiquitously present in all forms of life and are crucial for protein folding, complex formation, protein transport and sorting, cell cycle regulation, and signaling processes [202]. The HSP90 molecular chaperone was identified to protect client proteins from misfolding and degradation [203]. Likewise, HSP90 binds to the HIF-1α PAS domain, and HSP90 inhibitors induce proteasomal degradation of HIF-1α even independently of pVHL during HIF-1α modulation [204]. Moreover, it was verified that CORM-2 increases the HIF-1α/HSP90 interaction to promote HIF-1α’s stability [205], while it was found to compete with HSP90 for binding to HIF-1α and promote HIF-1α degradation [204]. Meanwhile, O_2_-independent degradation of HIF-1α is regulated by the competition of RACK1 and HSP90 for binding to HIF-1α. After the dephosphorylation of RACK1 by calcineurin, the HIF-1α degradation is reversed [206]. *O*-carboranylphenoxyacetanilide, a chemical that binds molecularly to HSP60, can inhibit the folding activity of HSP60-HSP10 and the ATPase activity of HSP60, serving as an inhibitor of HIF-1α [207, 208]. It is indicated that HSP60 could act as a protector factor for HIF-1α, but the underlying mechanism has not been completely unveiled. In addition, a recent study showed that HSP70 stabilization inhibited ferroptosis via HIF-1α SUMOylation, inducing lung cancer recurrence after insufficient radiofrequency ablation [209].

## HIF-1-mediated cellular response under normoxia

### Metabolic reprogramming and Warburg effect

As early as the 1920s, Otto Heinrich Warburg (the winner of the 1931 Nobel Prize in Physiology or Medicine) found that hypoxic tissue cells are more likely to become cancerous than normal tissue cells [210]. Warburg first proposed the metabolism that glucose can be converted to lactate even under oxygen‐abundant conditions, called “aerobic glycolysis” or the “Warburg effect” [211, 212]. It has been widely recognized that glycolysis releases much less energy than oxidative phosphorylation, but aerobic glycolysis, which provides less ATP, is preferred for rapidly proliferating tumor cells instead of oxidative phosphorylation [213]. In addition, metabolic intermediates of aerobic glycolysis also play an important role in cell anabolism and the maintenance of a cancer-promoting lactic acid microenvironment [214, 215]. The Warburg effect has been used in the auxiliary examination process of positron emission tomography/computed tomography (PET/CT). Owing to the Warburg characteristic metabolism of malignant tumor cells, glucose is in great demand, and when labeled by the positron nuclide ^18^F, namely using ^18^FDG, it can be detected by PET to form an image [216–218]. Previously, several studies have documented the transcriptional regulators of aerobic glycolysis in cancers, such as HIF-1α, c-MYC, p53, and ENO1 [219–223]. However, the specific mechanism underlying the regulatory molecules of the Warburg effect remains an alluring mystery.

An intricate connection has been shown between HIF-1 and the Warburg effect. HIF-1 as a transcriptional factor is regarded as being involved in the metabolic reprogramming, encoding glucose transporters (GLUT1, GLUT3) and glycolytic enzymes (ALDOA, ENO1, GAPDH, HK1, HK2, PFKL, PGK1, PKM2, LDHA) that convert glucose to lactate [224]. The stabilization of HIF-1α under normoxia is coincidentally coherent to the aerobic features of the Warburg effect, which also features in the metabolic transformation in cancer cells. Intriguingly, some of these glycolytic enzymes in turn regulate the activity of HIF-1, forming apparent positive feedback in the process. Lactate, the once anonymous end-product of “aerobic glycolysis,” provides fuel to neighboring cancer cells and facilitates metastasis and immunosuppression in the TME, which together enable cancer progression [223, 225]. As reviewed above, lactate has been shown to stabilize HIF-1α, seemingly through different mechanisms. In addition to the byproduct lactate, PKM2 as the downstream glycolytic enzyme of HIF-1 is a regulatory channel factor that can interact with HIF-1α and stabilize HIF-1α to stimulate further metabolic reprogramming [226].

### Cell metastasis

Metastasis and invasion are a multistep process and defined as a secondary factor that influences malignant growth at a distance away from the primary site of cancer [227]. HIFs are vital at all key stages of metastatic dissemination, for example, processes such as local migration within the tumor and invasion of the surrounding stromal tissue by inducing an EMT-like process and remodeling the extracellular matrix [228]. However, it seems that most studies have mainly focused on hypoxia signaling but have ignored HIFs’ regulatory function in normoxia. In several studies, WDR5 promoted HIF-1α activity and HIF-1α was reported to be an important effector in WDR5-induced cholangiocarcinoma metastasis under 95% air and 5% CO_2_ instead of hypoxia treatment [69]. Besides, UCHL1 has been shown to promote metastases in a HIF-1-dependent manner, in both normoxia and hypoxia, because of its deubiquitinating effect [174]. HDAC2 is reported to promote cell migration/invasion through HIF-1α stabilization in human oral squamous cell carcinoma [161]. Similarly, USP14 maintains HIF-1α stabilization via its deubiquitination activity in hepatocellular carcinoma (HCC). To elucidate, USP14 enhances cell migration and invasion in HCC cells, and subsequent rescue assays demonstrated that the inhibition was partially reversed by HIF-1α [170].

### Cell cycle regulation, proliferation, and cell death

Cell cycle is regulated by checkpoint proteins such as cyclins, cyclin-dependent kinases (CDKs), and cyclin-dependent kinase inhibitors. p53 is a crucial component of the surveillance mechanisms that halt cell cycle progression under adverse conditions and is involved in hypoxia-induced apoptosis [164, 229]. A HIF-1α-mediated cell cycle response could be found since HIF-1α interacts with p53 [101, 164], although this interaction emphasizes the considerable concentration of HIF-1 in cells under hypoxia. Additionally, activated HIF-1 causes a global slowdown of cell cycle progression at phases G1, S, and G2, leading to the loss of mitotic cells independent of p53 under normoxia [230]. Contrariwise, CDK1 and CDK2 have been shown to regulate lysosomal degradation (the lysosomal system and proteasome pathway are the two most significant degradation pathways) of HIF-1α to promote cell-cycle progression under hypoxia [231] [232]. Also, the definition of “resistant cancer cell populations” based on cell cycle heterogeneity is emphasized in cancer therapy [233]. Regarding cell proliferation, HIF-1α stabilization by USP14 is shown to impede cell proliferation under normoxia [170]. Additionally, HIF-1α SUMOylation via HSP70 knockdown inhibits A549 cell proliferation and ferroptosis after heat treatment [209]. HIF-1α’s instability via abrogation of PKM2 is shown to result in impaired proliferation and augmented apoptosis in pancreatic cancer cell lines [226].

**Fig. 4:**
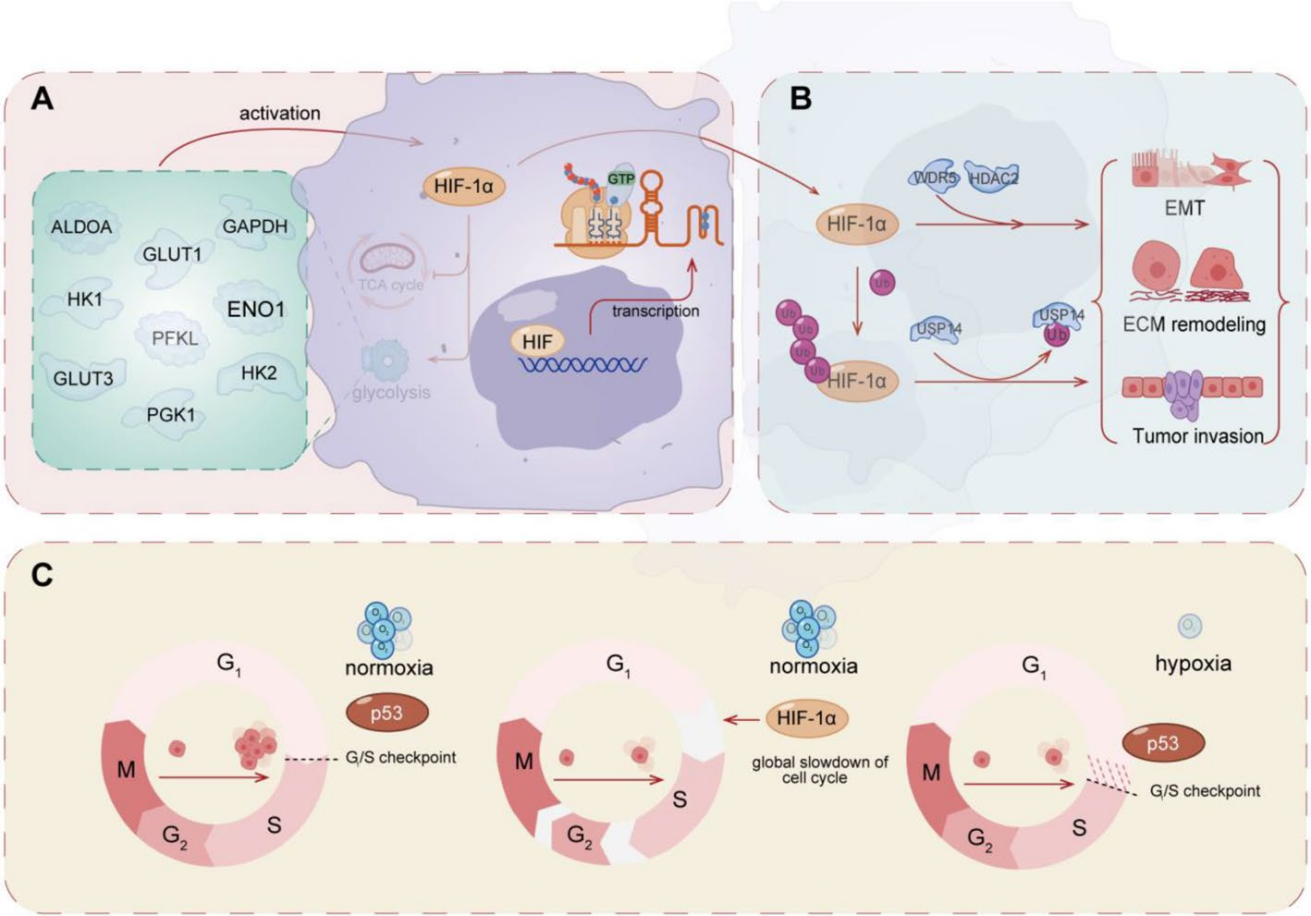
HIF-1-mediated cellular responses under normoxia. **A** HIF-1 contributes to metabolic reprogramming and the Warburg effect by encoding glucose transporters (GLUT1, GLUT3) and glycolytic enzymes (ALDOA, ENO1, GAPDH, HK1, HK2, PFKL, PGK1, PKM2, LDHA). **B** HIF-1 is involved in cellular metastasis through processes such as EMT, ECM remodeling, and tumor invasion. WDR5 and HDAC2 can enhance cell migration and invasion by stabilizing HIF-1α. Additionally, UCHL1 and USP14 maintain HIF-1α stability through their deubiquitination activity, promoting cellular migration. **C** HIF-1 plays a role in regulating the cell cycle, cell proliferation, and cell death: (i) in normoxia, the absence of p53 results in no checkpoint arrest, leading to normal proliferation; (ii) in pseudohypoxia, HIF-1α shortens the cell cycle independently of p53, as evidenced by reduced durations of various phases in the cycle, resulting in decreased cell proliferation; (iii) in hypoxia, p53 exerts its influence at the G1/S checkpoint, resulting in decreased cell proliferation

These cellular responses are shown in Fig. 4.

## HIF-1-related therapy in cancer

HIFs play crucial roles in both cancer and cardiovascular diseases and serve as a bridge between them. Specifically, HIF-1 is involved in protective mechanisms associated with conditions such as coronary artery disease, peripheral arterial disease, wound healing, organ transplant rejection, and colitis. On the contrary, HIF activity contributes to disease progression in hereditary erythrocytosis, pulmonary arterial hypertension, obstructive sleep apnea, and cancer [234]. In recent years, cardio‐oncology, a new clinical frontier, has reported cardiovascular toxicity related to cancer treatment. It must be pointed out that this review mainly emphasizes the involvement of HIFs in cancer. HIF-1 are important at various stages of cancer development, including angiogenesis, metastasis, metabolic reprogramming, invasion, EMT, and cell proliferation and survival [235]. Consequently, numerous clinical and experimental studies have identified HIFs as potential targets for cancer therapy and prognostic biomarkers. Elevated levels of HIF-1α are often related to poor clinical outcomes. For instance, high expression of HIF-1α has been associated with unfavorable responses to treatment in hepatocellular carcinoma undergoing local ablation therapies, as well as in oral cancer and locally advanced head and neck squamous cell carcinomas treated with cisplatin and radiation therapy [236–238]. Moreover, recent research indicates that HIF-1α expression in tumor-associated macrophages serves as an independent prognostic factor in kidney cancer. High levels of HIF-1α in this context are correlated with higher tumor grades, increased metastasis, and reduced overall survival. In contrast, elevated levels of HIF-2α are associated with lower tumor grades, decreased metastasis, and improved overall survival [239]. Additionally, chemoresistance and radioresistance are related to tumor relapse, progression, and metastasis, ultimately leading to worse overall survival rates [240, 241]. The stabilization of HIF-1α has been shown to contribute to chemoresistance and radiosensitization in various cancer types [118, 173, 242, 243]. In this section, we categorize the HIF-1-related drugs applied in cancer therapy, as summarized in Table 1.

**Table 1:** HIF-1-related drugs applied in cancer therapy

HIF1’s regulatory mechanism	More detailed mechanism	Agents	Current study phase	Clinical trial identifier	Refs.
Transcriptional level	NS	Alvocidib (Flavopiridol)	II	NCT00445341	[244, 290]
Translational level	Antisense oligonucleotide	EZN-2968	I	NCT01120288 NCT02564614	[252, 291, 292]
		RX-0047	None	NS	[293]
	Blocking mTOR/p70S6K/4E-BP1 pathway	Silibinin	II	NCT00487721 NCT01129570 NCT06187220	[249, 294, 295]
		KC7F2	None	NS	[296]
	PI3K inhibitors	Buparlisib	III	NCT04338399	[297, 298]
	Blocking mTOR	Rapamycin	Approved	NCT03500367	[299]
		Everolimus	Approved	NCT02842749	[300]
		Temsirolimus/CCI-779	Approved	NCT03500367	[301]
	ATR inhibition	AZD6738	II	NCT05514132	[302–304]
		VX-970 (M6620)	II	NCT03718091	[305, 306]
	Microtubule depolymerization	2ME2 and ENMD-1198	II	NCT00592579 NCT00444314	
	Inhibiting HIF-1α mRNA translation	2-Phenethyl isothiocyanate	II	NCT00691132	[307]
	Inhibiting Na^+^/K^+^ ATPase	Digoxin	I/II	NCT06030622	[308, 309]
PHDs	Possibly zinc deprivation	Metallothioneins (MTs)	None	NS	[253]
	Promoting the interaction between HIF1 and PHD	GYY4137	None	NS	[254, 310]
Ubiquitination	Related to proteasome	Cetuximab	Approved	NCT00044863 NCT00392769	[255]
	Inducing pVHL expression	LW6	None	NS	[311]
	Increasing HIF-1a and pVHL interaction	Wondonin	None	NS	[312]
Trans-activation	Hsp90 inhibition	Lonafarnib	III	NCT00773474 NCT00050336	[257]
		GA (geldanamycin) and analogs/tanespimycin	II	NCT00093405 NCT00087386	[258, 313]
		Ganetespib	III	NCT01562015 NCT01039519	[314, 315]
		Apigenin	II	NCT00609310	[316]
		Radicicol and its derivatives	None	NS	[317, 318]
	HDAC inhibition	SAHA and FK228 (vorinostat and romidepsin)	Approved	NCT00801151	[261, 318]
		Trichostatin A (TSA)	I	NCT03838926	[319]
		LAQ824	None	NS	[320]
	Inhibiting FIH-1 or the p300 recruitment	Bortezomib	Approved	NCT00512798	[262]
		Amphotericin B	None	NCT01615809	[263]
		Chetomin	None	NS	[321]
	Disturbing HIF1-HRE binding	Anthracycline	Approved	NCT04182568	[264]
		Polyamides	None	NS	[322]
		Echinomycin (NSC-13502)	None	NS	[323, 324]
	Inhibiting the recruitment of RNA polymerase II	Metformin	None	NCT05921942 NCT03684707	[265, 266]
	Disturb the dimerization of HIF-1α and HIF-1β	Anti-HIF-1a VHH and hetero-bivalent VHH nanobodies (AG1-5)	None	NS	[267]
		Acriflavine	None	NS	[268, 325]
Multitarget medicines	Inhibiting transcriptional initiation of the HIF-1α gene Suppressing transactivation activity of HIF-1α protein Disturbing the dimerization of HIF-1α and HIF-1β	Aminoflavone (AF)	II	NCT01015521	[326, 327]
	HIF-1α translation HIF-1α protein accumulation HIF-1 transcriptional activity	Camptothecin analogs (TPT, EZN-2208)	II	NCT01601184 NCT01036113	[328–330]
	Increasing pVHL activity Activating PHDs’ activity Impairing HIF-1 protein translation	Melatonin, NB-5-MT	None	NCT04137627 NCT04530097	[331–333]

### Targeting HIF-1α transcription

*Alvocidib (flavopiridol)* Alvocidib, a synthetic flavone, is derived from alkaloids found in the leaves and stems of the Indian plants *Amoora rohituka* and *Dysoxylum binectariferum*. As an inhibitor of CDKs, Alvocidib plays a role in cell cycle arrest, apoptosis, and various other cellular processes [244]. The most recent phase 2 clinical trial involving Alvocidib focused on newly diagnosed acute myeloid leukemia (AML) (clinicaltrials.gov identifier: NCT01349972). This trial compared the efficacy of Alvocidib combined with cytarabine and mitoxantrone (referred to as FLAM) versus the standard regimen of cytarabine and daunorubicin (known as 7 + 3). The results indicated that the FLAM regimen provided better event-free survival for the overall cohort and improved overall survival for patients under 50 years old [245, 246], suggesting that younger patients had a higher likelihood of achieving complete remission with FLAM compared with the 7 + 3 regimen. Despite these promising results, early clinical trials of Alvocidib as a single agent did not show significant therapeutic efficacy. However, further exploration of Alvocidib in combination therapies holds potential for future treatment advancements.

### Targeting HIF-1α translation

#### PI3K inhibition

*Buparlisib (BKM120)* As the most clinically advanced agent of all PI3K-inhibiting medicines, Buparlisib is a potent, selective, orally bioavailable class I pan-PI3K inhibitor with properties against p110-α, -β, -δ, and -γ enzymes at IC_50_ values of 52 μM, 166 μM, 116 μM, and 262 μM, respectively [247]. Given that PI3K operates upstream of the mTOR signaling pathway, it plays a crucial role in cellular translation processes. Two phase 3 clinical trials sponsored by Novartis Pharmaceuticals were completed to evaluate Buparlisib in breast cancer. One trial was conducted to verify the effect of Buparlisib in BELLE-4 with consistent paclitaxel treatment (clinicaltrials.gov identifier: NCT01572727), while the other was designed to test the effect of Buparlisib in BELLE-2 with consistent fulvestrant treatment (clinicaltrials.gov identifier: NCT01610284). In the BELLE-4 study, the combination of buparlisib and paclitaxel did not demonstrate an improvement in progression-free survival (PFS) compared with paclitaxel alone, both in the overall patient population and in those with PI3K pathway activation. Ultimately, the trial was halted at the end of phase II owing to lack of efficacy [247]. Conversely, in the BELLE-2 study, the combination of PI3K inhibition and endocrine therapy proved effective for postmenopausal women with hormone receptor-positive, HER2-negative advanced breast cancer that was resistant to endocrine treatment [248]. Although overall survival results favored the buparlisib and fulvestrant combination over the placebo plus fulvestrant, the difference was not statistically significant, and the combination was associated with a higher incidence of grade III/IV adverse events. Despite the mixed outcomes from these trials, ongoing investigation into Buparlisib remains promising, particularly regarding its potential use in combination therapies for specific subgroups of patients with breast cancer.

#### mTOR blocking

*Rapamycin (and its derivatives: Everolimus, Temsirolimus)* Commonly known as an mTOR inhibitor, rapamycin has been approved by the Food and Drug Administration (FDA) for treatment of RCC [248]. mTOR signaling plays a significant role in regulating the 5′-terminal oligopyrimidine tract (5′-TOP) of HIF-1α mRNA, which is crucial for controlling translational initiation. Increased mTOR activity often results in higher HIF-1α levels, promoting cell proliferation [50]. By inhibiting mTOR, rapamycin reduces HIF-1α mRNA translation and suppresses tumor growth. Ongoing research is exploring its potential in treating other diseases and in various combination therapies, as detailed in clinicaltrials.gov.

*Silibinin* Silibinin is a polyphenolic flavonolignan extracted from milk thistle (*Silybum marianum*), known for its antioxidant properties. It has been approved for the treatment of toxic liver damage, acute and chronic hepatitis, and abnormal liver function in fatty liver disease. Research indicates that silibinin can suppress HIF-1α accumulation, which is associated with significant dephosphorylation of the mTOR and its downstream effectors, such as p70S6K and eukaryotic initiation factor 4E-binding protein-1 [249]. This pathway is critical for regulating HIF-1α expression at the translational level, highlighting silibinin’s potential role in modulating cellular responses related to hypoxia and proliferation.

#### ATR inhibition

*Ceralasertib (AZD6738) and VX-970 (M6620)* Currently, these two inhibitors are undergoing phase I and II clinical trials, both as monotherapy and in combination with chemotherapy regimens. Ataxia telangiectasia and Rad3-related kinase (ATR) is a serine/threonine kinase belonging to the PIKK family, essential for DNA damage response signaling. Replicative stress induced by hypoxia activates ATR, thereby enhancing the translation of HIF-1α and augmenting its DNA-binding ability [250]. Meanwhile, ataxia telangiectasia mutated (ATM)-deficient tumors show heightened sensitivity to ATR inhibitors, highlighting the synthetic lethality approach where ATR inhibition can selectively target tumors with ATM mutations [251]. By inhibiting ATR, there is a reduction in HIF-1α expression and suppression of downstream genes such as GLUT1 and carbonic anhydrase IX, which are critical in the cellular adaptation to hypoxia [250].

#### Antisense oligonucleotides (ASOs)

*EZN-2968* EZN-2968 is an antisense oligonucleotide designed to bind specifically to complementary RNA sequences. ASOs work by pairing with target RNA molecules, thereby interfering with gene expression in various ways, including blocking translation and promoting RNA degradation. In pilot trials, EZN-2968 has demonstrated the ability to reduce both HIF-1α protein levels and the mRNA levels of several associated target genes. This suggests a potential therapeutic role for EZN-2968 in conditions where HIF-1α plays a critical role in tumor progression and survival [252]. The compound has undergone evaluation in two phase I clinical trials focusing on advanced solid tumors and lymphoma, including patients with liver metastases (clinicaltrials.gov identifier: NCT02564614). However, the current status of both studies is listed as “No results posted,” indicating that data from these trials have not yet been made publicly available. Further updates will be essential to understand the efficacy and potential applications of EZN-2968 in cancer therapy.

### Targeting PHDs

*Metallothionein* Metallothioneins are a group of low-molecular-weight proteins enriched in cysteine, known for their ability to bind metal ions and regulate their metabolism and distribution in the body. One proposed mechanism by which metallothioneins exert their effects is through zinc deprivation, influencing cellular processes that may be relevant in cancer treatment. Although metallothioneins have demonstrated promise in animal models and in vitro studies [253], clinical research exploring their potential as therapeutic agents remains limited. This lack of extensive investigation may be attributed to concerns surrounding the regulation of metal ion homeostasis and the associated safety implications. It is necessary to address these challenges, which is essential for unlocking the therapeutic potential of metallothioneins in oncology.

*GYY4137* GYY4137 has been shown to enhance the interaction between HIF-1 and PHD2, facilitating the degradation of HIF-1α. This process occurs through the persulfidation of PHD2 at specific cysteine residues within its zinc finger domain, which strengthens the binding between PHD2 and HIF-1. As a result, this interaction promotes the hydroxylation and subsequent degradation of HIF-1. As a slow-releasing hydrogen sulfide donor, GYY4137 has exhibited promising anticancer effects in both in vitro and in vivo studies [254]. Nevertheless, despite these findings, there has yet to be any clinical research conducted on GYY4137. Further investigations are necessary to explore its therapeutic potential in clinical settings.

### Related to ubiquitination

*Cetuximab (Erbitux)* Cetuximab is a chimeric monoclonal antibody that targets and inhibits the epidermal growth factor receptor (EGFR). Originally developed for treating metastatic colorectal cancer, it has also received FDA approval for head and neck squamous cell carcinoma (HNSCC) and colorectal cancer. Research has shown that cetuximab can decrease the cellular levels of HIF-1α when used in conjunction with the proteasome inhibitor lactacystin. This finding suggests that cetuximab primarily influences protein synthesis, leading us to classify it within the context of ubiquitination owing to its relationship with the proteasomal degradation pathway [255]. Further understanding of cetuximab’s mechanisms may enhance its therapeutic application in oncology.

### Targeting trans-activation

#### Hsp90 inhibition

*Lonafarnib (SCH66336)* Lonafarnib, initially developed as a non-peptidomimetic inhibitor of farnesyl transferase, targets an enzyme responsible for the posttranslational lipid modification of various proteins related to disease pathways, including cancer and progeria [256]. In 2020, lonafarnib received FDA approval for the treatment of progeria, highlighting its therapeutic potential. Recent research has revealed that lonafarnib (SCH66336) also inhibits angiogenic activities in non-small cell lung cancer and HNSCC. This inhibition is primarily achieved through two mechanisms: (1) by reducing the expression of HIF-1α stimulated by hypoxia or insulin-like growth factor and (2) by disrupting the interaction between HIF-1α and Hsp90. This disruption promotes the proteasomal degradation of HIF-1α, leading to decreased production of VEGF, a key factor in angiogenesis [257]. While several clinical trials exploring lonafarnib’s efficacy in cancer have been discontinued, there remains considerable opportunity for further investigation. Continued research into lonafarnib’s mechanisms may uncover additional applications in oncological therapies targeting the HIF-1α pathway.

*Geldanamycin and tanespimycin* Geldanamycin, an inhibitor of Hsp90, has been shown to promote the ubiquitination and subsequent proteasomal degradation of HIF-1α in RCC cells, regardless of oxygen levels [258]. Despite these promising preclinical results, phase II clinical trials have yielded mixed outcomes. Specifically, one trial reported that 17-AAG, a derivative of geldanamycin, did not produce objective responses in patients with clear cell or papillary renal cell carcinoma at the administered dose and schedule [259]. Moreover, another trial (clinicaltrials.gov identifier: NCT00087386) was terminated before conclusions could be drawn.

#### HDAC inhibition

*Vorinostat (SAHA) and romidepsin (FK228)* SAHA, a synthetic hydroxamic acid derivative, inhibits HDACs. It suppresses the HIF pathway by enhancing the degradation of the HIF-1α molecule and has demonstrated promising results in studies involving solid tumors and hematologic malignancies [260, 261]. Vorinostat (SAHA) and romidepsin (FK228) were approved for the treatment of cutaneous T cell lymphoma. The application for other tumors is still under investigation.

#### Inhibiting FIH-1 or the p300 recruitment

*Bortezomib (Velcade)* Bortezomib (PS-341), a proteasome inhibitor, has been evaluated for treating multiple myeloma and various solid tumors. Clinical studies suggest that bortezomib’s ability to inhibit tumor angiogenesis and hypoxic adaptation is associated with its repression of HIF-1α transcriptional activity. This effect is achieved by enhancing the FIH-mediated inhibition of p300 recruitment [262].

*Amphotericin B* Amphotericin B effectively treats severe fungal infections such as candidiasis, histoplasmosis, cryptococcal meningitis, and aspergillosis. However, its long-term use often results in adverse effects, notably anemia because of suppression of EPO. Researchers found that amphotericin B blunts EPO response to hypoxia by reinforcing FIH-mediated repression of HIF-1 [263].

#### Disturbing HIF-1-HRE binding

*Anthracycline* Anthracycline chemotherapeutic agents, which are structurally similar topoisomerase inhibitors, are commonly used to treat various tumors. Doxorubicin (DXR), a member of the anthracycline class, has been reported to impair the transcriptional response of the HIF by inhibiting the binding of the HIF heterodimer to the consensus –RCGTG– enhancer element [264]. DXR has been approved by the FDA for the treatment of breast cancer, acute myeloid leukemia, acute lymphoblastic leukemia, and other conditions, usually in combination with other drugs.

#### Inhibition of recruitment of RNA polymerase II

*Metformin* Metformin, originally developed as an oral medication, is widely used to treat type 2 diabetes, to lower blood glucose levels by improving insulin sensitivity, to reduce hepatic glucose output, and to enhance muscle glucose uptake. Metformin inhibits mitochondrial complex I activity, thereby decreasing mitochondrial oxygen consumption, promoting cytosolic oxidation, and inducing the proteolysis of HIF-1 protein [265]. A current study reveals that short-term treatment with the standard diabetic doses of metformin does not decrease tumor proliferation in women suffering from endometrioid endometrial cancer who are awaiting hysterectomy [266].

#### Disturbing the dimerization of HIF-1α and HIF-1β

*AG1-5 (anti-HIF-1a VHH and hetero-bivalent VHH nanobodies)* AG1-5 is a novel anticancer drug that acts as an inhibitor of HIF-1α by binding to HIF-1α and preventing its dimerization with HIF-1β, thereby inhibiting the HIF-1 signaling pathway [267]. This pathway is often overactivated in various tumors, driving tumor growth and metastasis. The mechanism of AG1-5 makes it a promising candidate for cancer therapy, particularly for cancers where inhibition of the HIF-1 signaling pathway is desired. However, there are currently no clinical trials available for AG1-5.

*Acriflavine* Acriflavine is a synthetic dye and antiseptic that has been used historically for its antibacterial and antifungal properties. It is commonly used in laboratory settings for staining purposes and has also been investigated for its potential therapeutic applications, including its effects on cancer and bacterial infections [268]. However, there are currently no clinical trials available for acriflavine. Despite its historical use as an antiseptic and its potential applications in various fields, such as oncology and microbiology, there has been a lack of recent clinical research and studies to explore its efficacy and safety in contemporary medical practice. The absence of clinical trials limits our understanding of its current therapeutic potential and its role in modern medicine.

## HIF-1-related therapy in cardio-oncology

The incidence and mortality of tumors are increasing year on year, as are cardiovascular diseases [269, 270]. As our understanding of the molecular mechanisms underlying these diseases deepens, the focus of research has shifted toward exploring the shared pathophysiological processes between cancer and cardiovascular diseases. Meanwhile, discussion of the co-pathogenesis between tumor and cardiovascular disease has become a new spotlight. There is growing evidence that the progression of several cancers is related to the development of cardiovascular diseases on the biochemical and molecular levels [271]. It is found that HIF signaling is involved in cancer and cardiovascular diseases, and HIF-1 is important in the process of disease development [272]. For example, an early in vitro study demonstrated that inactivation of PHD2, which increased HIF-1 activity, could promote vessel normalization and mitigate potential cardiotoxicity associated with cancer treatments [273]. Conversely, cardiovascular diseases also contribute to increased cancer mortality, as indicated by a recent prospective cohort study [274]. The interconnection between cancer and cardiovascular diseases has led to the emergence of cardio-oncology, an expanding area of clinical frontiers. To further deepen this field, a new classification system, known as the “cardio-oncology syndrome” (COS), has been proposed [275]. COS can be categorized into five classifications, and COS type II encompasses underlying mechanisms through which cancer treatments can lead to cardiovascular disease [275]. To avoid terminological confusion and focus on the main points, this review primarily discusses COS type II, with an emphasis on approved drugs and their therapeutic implications.

Rapamycin and its derivatives, temsirolimus as well as everolimus, are well-established mTOR inhibitors that have been widely used in oncology since the early 2000s. Although no clinical trials or studies have directly linked rapamycin or its derivatives to cardiac toxicity in cancer patients, these agents are associated with an increased risk of hyperglycemia, hyperlipidemia, and hypertension—well-known risk factors for cardiovascular diseases [276]. One of the most discussed side effects of mTOR inhibitors is dyslipidemia. For instance, assessing adjuvant everolimus after surgery for RCC in a double-blind randomized controlled trial, hypertriglyceridemia (53%) and hypercholesterolemia (49%) were laboratory abnormalities most frequently observed [277]. Everolimus is commonly used in drug-eluting stents (DES) to prevent re-atherosclerosis following stent implantation despite the potential for dyslipidemia, which highlights the role of local microenvironment modulation in cardiovascular outcomes. Interestingly, blood concentrations of everolimus in DES have been shown to be lower than those required for safe oral administration, as demonstrated in a previous study [278]. The difference in drug concentration could explain the apparent contradiction between the dyslipidemic effects observed in systemic therapy and the beneficial effects of everolimus in DES. Furthermore, inhibition of HIF-1 has been reported to improve dyslipidemia and reduce atherogenesis by decreasing adipose ceramide production in animal models [279]. This seemingly contradictory finding suggests the existence of additional promising mechanisms through which mTOR inhibitors, particularly everolimus, may exert both beneficial and adverse cardiovascular effects.

Cetuximab, an EGFR monoclonal antibody, is commonly used in the treatment of various cancers. To date, there has been no clear association between cetuximab and common cardiotoxicities. However, cardiac arrest was observed in 10% (3 out of 30) of patients with advanced solid tumors receiving a dose of 12 mg/kg in a dose-escalation trial of Sym004 (a 1:1 mixture of cetuximab and panitumumab), which was regarded as an incidental finding [280]. In another meta-analysis, cardiopulmonary arrest was reported as a rare adverse event, but it did not obtain significant attention as a major concern [281].

Vorinostat and romidepsin exert their effects primarily in hematologic malignancies by inhibiting HDACs, and the potential contribution of vorinostat to arrhythmias remains unclear. However, a case report described the occurrence of torsades de pointes (a specific form of ventricular arrhythmia) following vorinostat treatment, with the authors suggesting that the drug may have been a contributing factor [282]. A previous study found that a single supratherapeutic dose of vorinostat did not prolong the QTc interval in patients with advanced cancer [283]. This discrepancy indicates the need for more prospective and well-designed studies to clarify the potential arrhythmic risks of vorinostat. Additionally, a study identified preprocedural serum levels of HIF-1 and VEGF as potential biomarkers for the risk of reperfusion arrhythmia following percutaneous coronary intervention [284]. Notably, HIF-1 has also been related to atrial fibrillation, a common atrial arrhythmia [285]. These findings suggest that HIF-1 plays a role in the arrhythmic toxicity of vorinostat, making it important to carry out further research into the drug’s cardiovascular effects.

Bortezomib is a selective, reversible proteasome inhibitor primarily used for the treatment of multiple myeloma. Its potential cardiotoxicity has been reported, with adverse effects including heart failure, arrhythmias, and accelerated atherosclerosis [286]. A mouse study suggested that targeting arginase 1 could help to mitigate the cardiotoxic effects of bortezomib [287]. Additionally, a study has shown that the expression of HIF-1α in bone marrow endothelial cells is associated with disease relapse and drug resistance in multiple myeloma patients, and it represents a promising therapeutic target [288]. Despite these findings, research on the role of HIF-1α in the context of cardio-oncology in multiple myeloma is limited. Further investigation in this area is warranted to better understand the underlying mechanisms and potential therapeutic interventions.

Anthracycline is a well-established chemotherapy agent, widely recognized for its cardiovascular toxicity, which includes heart failure, arrhythmias, and myocardial infarction [289]. Mechanistically, anthracycline exerts its effects by inhibiting topoisomerase IIα and generating ROS. However, the precise properties of its cardiotoxic effects remains a subject of dispute, with several studies providing conflicting results [272]. These controversies have been discussed in the context of hypoxia signaling and oxygen metabolism in cardio-oncology, highlighting the need for further exploration of the underlying mechanisms.

## Discussion and conclusion

HIF-1 is a highly sensitive molecule that can be regulated and reversed by multiple mechanisms, even under normoxic conditions. One particularly intriguing and complex aspect of HIF-1 research is its interaction with ROS under normoxia. ROS are known to be involved in a wide range of biological processes, including angiogenesis and apoptosis, and play a significant role in various pathophysiological conditions such as inflammation, cancer, and cardiovascular diseases. The interplay between HIF-1 and ROS in normoxia offers valuable insights into these processes, highlighting a critical area for further investigation. Furthermore, when discussing normoxia in the context of oncology, the Warburg effect is a central concept, as it represents a hallmark of cancer cell metabolism. In this review, we comprehensively categorize the regulatory mechanisms of HIF-1 under normoxia, emphasizing its role as a key molecule that bridges the Warburg effect and pseudohypoxia. We underline that HIF-1 is not merely a passive regulator but plays an adaptive role in the metabolic and pathophysiological alterations associated with cancer.

Targeting HIF-1 is promising, for its critical role in angiogenesis, metabolic reprogramming, and cell survival. However, most of the HIF-1 inhibitors reported to date share a common limitation: a lack of specificity. This issue arises from two primary factors. First, agents that directly target HIF-1 transcription and translation often result in significant adverse drug events, hindering their progression to the next stage of clinical trials. Second, drugs that act on posttranslational pathways of HIF-1 can exert broader biological effects, leading to undesirable outcomes. Moreover, these off-target effects may be dose-dependent. Generating robust clinical evidence for HIF-1 inhibition is further complicated by these challenges. There may be two ways to solve the current situation. On the one hand, further drug design may need to become safer and more specific. On the other hand, side-effects of broad target drugs should be solved.

Cardio-oncology, an emerging field that has evolved alongside advances in cancer treatment, aims to extend patient survival while minimizing cardiovascular toxicities. In this context, this review highlights the side effects of HIF-1 inhibitors in clinical oncology, underscoring the need for early-phase clinical trial validation of these agents. Furthermore, it stresses the potential of combination therapies, which may offer more promising therapeutic outcomes for the clinical application of HIF-1 inhibitors. Despite progress in the field, only a limited number of studies have investigated the role of HIF-1, a key molecule in both cardiovascular diseases and tumor progression. Current cardio-oncology research primarily focuses on symptom management and addressing complications once they arise, rather than exploring the underlying molecular mechanisms. We propose that HIF-1 may play a pivotal role in cardio-oncology, particularly in the context of atherosclerotic plaque formation (owing to its involvement in metabolic pathways) and other related cardiovascular conditions. We argue that future cardio-oncology research should place greater emphasis on HIF-1, as this may help in the early identification of therapeutic agents and the development of strategies to prevent cardiotoxicity.

In conclusion, hypoxia signaling plays a critical role in cellular adaptation to low oxygen levels, with HIF-1 acting as a central regulator owing to its instability under normal oxygen conditions. However, numerous studies, both early and recent, have demonstrated that HIF-1α can remain stable even under normoxic conditions. This stability enables HIF-1α to continuously regulate downstream molecular pathways, a phenomenon that often contributes to malignancy. In this review, we explore alternative mechanisms that allow HIF-1α to remain stable outside of hypoxia and examine its role in the cascading processes associated with this stability. We then discuss key HIF-1α-mediated cellular responses under normoxia, emphasizing the associated metabolic reprogramming. Furthermore, we provide an overview of HIF-1-related drugs—both those approved and those still under clinical investigation—within the context of the previously outlined regulatory mechanisms. Given HIF-1’s crucial role in both cancer and cardiovascular diseases, emerging research suggests that HIF-1 may serve as an important link between oncology and cardiology—an area often referred to as cardio-oncology. In this context, HIF-1 may play a dual role by influencing tumor progression and metastasis, while also contributing to cardiovascular pathologies, such as ischemic heart disease and heart failure. As such, HIF-1’s stability in normoxia may have profound implications not only for cancer progression but also for cardiovascular health, offering a potential therapeutic target for both fields. Future research should focus on elucidating the precise mechanisms that allow HIF-1α to remain stable under normoxic conditions and exploring how this stability contributes to pathological states, particularly in the context of cardio-oncology. Our review clarifies the mechanisms underlying HIF-1α stabilization in normoxia, providing insights that could facilitate the development of targeted therapies. Optimizing existing therapies, whether as single agents or in combination, could provide promising strategies for treating diseases associated with aberrant HIF-1α activity, bridging the gap between cancer and cardiovascular disciplines.

## Data Availability

Not applicable.

## Abbreviations

ATM: Ataxia telangiectasia mutated
ATR: Rad3-related kinase
bHLH: Basic Helix-Loop-Helix
CBP: CREB binding protein
ccRCC: Clear cell renal cell carcinoma
CDKs: Cyclin-dependent kinases
Co^2^⁺: Cobalt
CODD: C-terminal oxygen-dependent degradation domain
C-TAD: C-terminal transactivation domain
DES: Drug-eluting stents
DUBs: Deubiquitinases
DXR: Doxorubicin
EAF2: ELL-associated factor 2
EGFR: Epidermal growth factor receptor
eIF4F: Eukaryotic initiation factor 4F
EMT: Epithelial-mesenchymal transition
EPO: Erythropoietin
Fe^2^⁺: Divalent iron
FH: Fumarase
FIHs: Factors inhibiting HIF
GDH: Glutamic dehydrogenase
GPT: Glutamic pyruvate transaminase
HATs: Histone acetyltransferases
HDAC: Histone deacetylase
HIFs: Hypoxia-inducible factors
HNSCC: Head and neck squamous cell carcinoma
HREs: Hypoxia-responsive elements
HSPs: Heat-shock proteins
ID: Inhibitory domain
IDH: Isocitrate dehydrogenase
KLF: Kruppel-like factor
LDH: Lactate dehydrogenase
MAPK: Mitogen-activated protein kinases
mTORC1: Mechanistic target of rapamycin complex 1
NF-κB: Nuclear factor kappa-light-chain-enhancer of activated B cells
Ni^2^⁺: Nickel
NODD: N-terminal oxygen-dependent degradation domain
N-TAD: N-terminal transactivation domain
ODDD: Oxygen-dependent degradation domain
p70S6K: P70S6 kinase
PAD: Peripheral arterial disease
PAH: Pulmonary arterial hypertension
PAS-A: Per-Arnt-Sim-A
PAS-B: Per-Arnt-Sim-B
PHD: Prolyl hydroxylase domain-containing protein
pVHL: Von Hippel–Lindau tumor suppressor protein
R-2-HG: R-2-hydroxyglutarate
ROS: Reactive oxygen species
RPS6: Ribosomal protein S6
S-2-HG: S-2-hydroxyglutarate
SDH: Succinate dehydrogenase
SP1: Specificity Protein 1
SIRT: Sirtuin
SUMO: Small ubiquitin-like modifier
TCA: Tricarboxylic acid
TFs: Transcription factors
TME: Tumor microenvironment
Trans-activation: Transcriptional activation
Ubl: Ubiquitin-like
UCHL1: Ubiquitin C-terminal hydrolase-L1
USPs: Ubiquitin-specific proteases
VACV: Vaccinia virus
VEGF: Vascular endothelial growth factor
αKG: α-Ketoglutarate
2OGDD: 2-Oxoglutarate-dependent dioxygenase
4EBP1: 4E-binding protein-1
